# αCGRP deficiency aggravates pulmonary fibrosis by activating the PPARγ signaling pathway

**DOI:** 10.1038/s41435-023-00206-x

**Published:** 2023-05-25

**Authors:** Xiaoting Lv, Qingquan Chen, Zewei Zhang, Kaili Du, Yaping Huang, Xingzhe Li, Yiming Zeng

**Affiliations:** 1 Department of Respiratory and Critical Care Medicine, the First Affiliated Hospital of Fujian Medical University, Fuzhou, 350005 China; 2 Department of Respiratory and Critical Care Medicine, National Regional Medical Center, Binhai Campus of the First Affiliated Hospital, Fujian Medical University, Fuzhou, 350212 China; 3 Institute of Respiratory Disease, Fujian Medical University, Fuzhou, 350005 China; 4 Department of Laboratory Medicine, School of Medical Technology and Engineering, Fujian Medical University, Fuzhou, 350004 China; 5 School of Medical Technology and Engineering, Fujian Medical University, Fuzhou, Fujian 350004 China; 6 Department of Pulmonary and Critical Care Medicine, the Second Affiliated Hospital of Fujian Medical University, Respirology Medicine Centre of Fujian Province, Quanzhou, China

**Keywords:** Cell death and immune response, Gene expression

## Abstract

In order to explore whether αCGRP (*Calca*) deficiency aggravates pulmonary fibrosis (PF). Clinical data from patients with PF (*n* = 52) were retrospectively analyzed. Lung tissue from a bleomycin (BLM)-induced rat model was compared with that of *Calca*-knockout (KO) and wild type (WT) using immunohistochemistry, RNA-seq, and UPLC-MS/MS metabolomic analyses. The results showed that decreased αCGRP expression and activation of the type 2 immune response were detected in patients with PF. In BLM-induced and *Calca*-KO rats, αCGRP deficiency potentiated apoptosis of AECs and induced M2 macrophages. RNA-seq identified enrichment of pathways involved in nuclear translocation and immune system disorders in *Calca*-KO rats compared to WT. Mass spectrometry of lung tissue from *Calca*-KO rats showed abnormal lipid metabolism, including increased levels of LTB4, PDX, 1-HETE. PPAR pathway signaling was significantly induced in both transcriptomic and metabolomic datasets in *Calca*-KO rats, and immunofluorescence analysis confirmed that the nuclear translocation of PPARγ in BLM-treated and *Calca*-KO rats was synchronized with STAT6 localization in the cytoplasmic and nuclear fractions. In conclusion, αCGRP is protective against PF, and αCGRP deficiency promotes M2 polarization of macrophages, probably by activating the PPARγ pathway, which leads to activation of the type 2 immune response and accelerates PF development.

## Introduction

Interstitial lung disease (ILD) collectively references several fatal lung diseases—each one classified as a rare disease—typically characterized by alveolitis and interstitial fibrosis [1]. Despite these common lung tissue phenotypes, little is known regarding the pathogenesis of ILD, and patients present in the clinic with highly heterogeneous symptoms [1]. Patients with ILD can develop pulmonary fibrosis (PF), which can induce respiratory failure; thus, PF is associated with progression of ILD and a median survival of only 3 years after diagnosis [2]. Recently, fibrosis in ILD has been divided into idiopathic pulmonary fibrosis (IPF) and progressive pulmonary fibrosis (PPF). Whereas neither subtype of fibrotic ILD can be reversed, the latter is more aggressive, and lung transplantation and oxygen therapy are among the only effective treatments [3, 4]. Therefore, there is an urgent unmet medical need to understand the pathogenesis of PF and to identify effective strategies to intervene in fibrotic ILD.

Calcitonin gene related peptide (CGRP) is a protein secreted by a variety of nerve and immune cells [5–7]. CGRP has complex biological functions such as vasodilation in the plasma [8] and pain signaling in the cerebrospinal fluid [9], and it is also involved in cell proliferation, differentiation, and apoptosis after injury [6, 7, 10, 11]. There are two human CGRP subtypes, αCGRP and βCGRP, which are encoded by *CT/CALCA* and *CALCB* respectively. Approximately 90% of circulating CGRP is the αCGRP subtype, and it is mainly distributed in the central and peripheral nervous systems [12–14]. βCGRP is found in the peripheral nervous system and in internal organs, including the intestine and thyroid [13, 15]. Recent studies have shown that, in the lung, CGRP promotes polarization of macrophages to the M2-phenotype and inhibits inflammation [16], and CGRP can also inhibit the differentiation of cardiac fibroblasts [17, 18]. Also, our group recently showed that decreased αCGRP expression in the lung tissues of *Calca*-knockout (*Calca*-KO) rats resulted in the development of fibrosis [19]. Based on this, we speculate that αCGRP deficiency contributes to PF, but this has not been confirmed, and there is currently no known mechanism by which αCGRP deficiency would induce fibrosis.

We evaluated lung tissues from patients with PF and from a classical model of bleomycin (BLM)-induced PF in rats, as well as from *Calca*-KO rats. We confirmed decreased αCGRP expression in human tissue and in both animal models compared to untreated wild type (WT) animals. By comparing fibrotic lung tissue of *Calca*-KO rats with that of BLM-induced rats and patients with PF, we confirmed that αCGRP affects nuclear translocation and PPARγ as well as regulates the PPAR pathway. Moreover, we demonstrate that αCGRP deficiency promotes collagen deposition and induces M2 macrophage polarization, probably by activating the PPARγ pathway, which leads to activation of the type 2 immune response and accelerates PF development. We thus identify αCGRP as a critical node in PF development and urge further studies to develop strategies to therapeutically target this pathway.

## Materials and methods

### Patient characteristics

This was a retrospective study that included patients hospitalized between August 2018 and June 2022 with IPF or PPF. Patients included in the retrospective analysis were diagnosed with ILD by chest CT scanning, sequential fluorine-18 fluorodeoxyglucose ((18) F-FDG) positron emission tomography (PET)/computed tomography (CT), and/or pathological examination. Distinction between PPF and IPF was made using previously defined comprehensive diagnostic criteria [4, 20]. Patients with malignant tumors, bronchiectasis, and/or pulmonary cystic fibrosis were excluded. A total of 52 patients were eligible and analyzed in the retrospective study. Clinical laboratory data from patient medical records were reviewed. A cohort of 12 patients with pulmonary bullae were analyzed as a control group. This study was approved under no. [2018]036 by the Medical Ethics Committee of the First Affiliated Hospital of Fujian Medical University (Fuzhou, Fujian, China). Informed consent for participation and publication of the case reports was obtained from all patients. Of the 52 patients, 6 patients (cases 1–6) had undergone CT-guided fine-needle aspiration biopsy of the lung lesions. For these patients, lung tissue samples stained with hematoxylin and eosin (H&E) for pathology were available for our review.

### Construction of *Calca*-KO rats

Shang Hai Model Organisms Co., Ltd, generated and validated *Calca*-KO rats. Briefly, heterozygous *Calca*^*+/−*^ rats were obtained using CRISPR/Cas9 technology and then homozygous animals were obtained and housed in a 1:1 or 2:1 sex ratio to mate. Female rats gave birth ~21 days after pregnancy and offspring were genotyped using tail tissue when they were between 7 and 10 days old. Confirmed homozygous *Calca*-KO rats were then separated into cages according to sex about 4 weeks after birth. Ten female rats, eight weeks old, were used in this experiment.

### Experimental PF models and grouping

All animal experiments were approved by the Experimentation Ethics Committee on Animal Rights Protection of Fujian Medical University Animal Experiment Center (Fujian, China). During the experiments, animals were treated in compliance with the “Guiding Opinions on the Ethical Treatments of Laboratory Animals” published by the Ministry of Science and Technology in 2006. All procedures strictly followed the National Institutes of Health guidelines for the care and use of laboratory animals. Twenty female Sprague–Dawley (SD) rats, eight weeks old and weighing 280–320 g, were housed in comfortable cages at temperatures between 22–25 °C with adequate water and food supply. Then rats were randomized to receive BLM 5 mg/kg or an equal volume of saline intratracheally. Twenty-eight days after treatment, all rats were sacrificed using an overdose of anesthesia and pulmonary tissues were harvested.

### Histological evaluation

Lungs were fixed in neutral formalin and embedded in paraffin for immunohistochemistry studies. Lung sections were stained with H& E and apoptosis markers using standard procedures. Briefly, the sections were deparaffinized in xylene (2 × 5 min) and rehydrated with successive 1-minute washes in 70%, 80%, 95%, and 100% ethanol. Then, they were stained with the relevant marker (2 min), rinsed with distilled water, 0.1% hydrochloric acid in 50% ethanol, and tap water for 15 min. Another stain-and-rinse cycle sometimes followed with a second marker. After that, the slides were dehydrated, treated with xylene (2 × 5 min), and mounted with cover slips.

### Immunohistochemical studies

Paraffin-embedded rat lung tissue sections were deparaffinized in xylene and rehydrated in graded ethanol washes. Antigen retrieval was performed by cooking tissue sections for 30 min in Tris-EDTA buffer and applying the following primary antibodies: CD68^+^ (ready-to-use, Maixin, Fuzhou, China), CD3^+^ (1:1000, Santa Cruz, California, USA), TGFβ1 (1:800, Bioworld, Louis Park, MN, USA), βCGRP (1:800, ABclonal, Wuhan, China), αCGRP (1:800, ABclonal, Wuhan, China), BAX (1:500, proteintech, Chicago, USA), and PPAR-ɤ (1:800, Bioss, Beijing, China) at 4 °C overnight. Horseradish peroxidase-conjugated secondary antibodies (Dako, Denmark) were used to probe the primary antibodies. Lung fibrosis lesions were classified according to their intensity by Masson’s trichrome into three categories.

### Immunofluorescence staining

Lung tissue sections were incubated with CD68^+^ (1:500, Abcam, Cambridge, UK), iNOS (1:500, Abcam, Cambridge, UK), CD206^+^ (1:500, Abcam, Cambridge, UK), PPARγ (1:100, Bioss, Beijing, China), or STAT6 (1:1000, ABclonal, Wuhan, China) primary antibodies overnight at 4 °C. After washing, the slices were incubated with goat anti-mouse IgG (H + L) secondary antibody–FITC conjugate (1:100, Boster, Wuhan, China) or goat anti-rabbit IgG (H + L) secondary antibody–Cy3 Conjugate (1:100, Boster, Wuhan, China) for 2 h at room temperature and then with 4′,6-diamidino-2-phenylindole (DAPI; Boster, Wuhan, China) for 10 min at room temperature. The slices were digitally photographed using a fluorescence microscope.

### Flow cytometry

Bead-based immunoassays were conducted using the same basic principles as sandwich immunoassays. Beads were differentiated by size and internal fluorescence intensities. Each bead set was conjugated with a specific antibody on its surface to capture a particular analyte. Then, a selected panel of capture beads was mixed and incubated with a sample containing target analytes specific to the capture antibodies. A biotinylated detection antibody cocktail was added to bind to its specific analyte immobilized on the capture beads, thus forming capture bead–analyte–detection antibody sandwiches. Subsequently, streptavidin-phycoerythrin (SA-PE) was added to bind the biotinylated detection antibodies, and this resulted in a fluorescence signal in which the intensity was proportional to the amount of bound analytes. Because the beads were differentiated by size and internal fluorescence intensity on a flow cytometer, analyte-specific populations could then be segregated and quantified using the PE fluorescence signal. Analyte concentrations were determined using a standard curve generated with the same assay.

### Transcriptome sequencing of rat lung tissues

Lung tissues from WT and *Calca*^+/−^ rats (*n* = 3 samples per group) were submitted for sequencing at Beijing Genomics Institute. DNA nanoballs (DNBs) were loaded into the patterned nanoarray and pair-end 100-base reads were generated using the BGIseq500 platform (BGI-Shenzhen, China). A total of 18,806 genes were detected. Each sample produced an average of 6.57 g data. The average comparison rate for the sample comparison genome was 91.79% for the comparison gene set was 66.48%.

### Lipid metabolism

Lung tissues from WT and *Calca*^+/−^ rats (*n* = 6 samples per group) were submitted to Personalbio Co., Ltd for testing. Target substance was obtained using repeat centrifugation to remove impurities and then solid-phase extraction was used to enrich for oxidized lipids, which were analyzed using a LC–MS/MS platform. In total, 120 species of oxidated lipids were detected, including downstream oxidative metabolites of arachidonic acid (AA), linoleic acid (LA), α-linolenic acid (ALA), docosahexaenoic acid (DHA), eicosapentaenoic acid (EPA), and dihomo-gamma-linolenic acid (DGLA). Data were acquired on an Ultra Performance Liquid Chromatography (UPLC) and Tandem Mass Spectrometry (MS/MS) and the database was constructed based on the standard. Quantitative analysis was performed using the multiple response monitoring mode of triple-quadrupole MS.

### Statistical analysis

All experiments were independently repeated three times. All experimental data are presented as mean ± standard deviation (SD) analyzed by SPSS 22.0 statistical software and graphed by Graphpad Prism 8.0 unless otherwise indicated. The independent sample Student’s *t* test was used for comparisons between the two groups satisfying the normal distribution and homogeneity of variance. Analysis of variance (ANOVA) was used for comparison of three or more groups. Single-factor ANOVA was used to test homogeneity of variance between groups. A nonparametric test was used when data were not normally distributed. *P* < 0.05 was considered statistically significant.

## Results

### Activation of type 2 immune responses in patients with PF

**Fig. 1:**
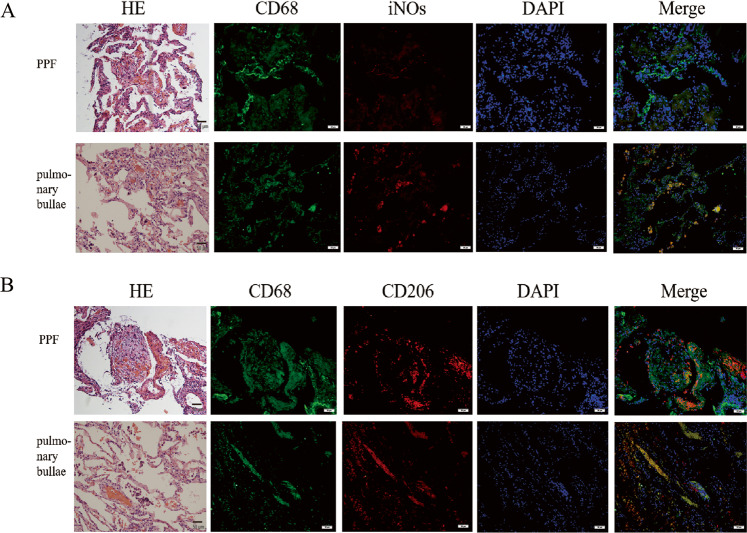
The polarization of M2 macrophages in patients with PF. **A** Immunofluorescence staining showed CD68^+^ iNOS M1 subtype macrophages in the lung tissues of pulmonary bullae patients were obviously more than that in PF patients (green represents CD68^+^ and red represents iNOS)(_200); **B** predominant CD68^+^CD206^+^M2 subtype differentiation was showed in the lung tissues of PF patients (green represents CD68^+^ and red represents CD206^+^)(_200).

We retrospectively analyzed clinical data from 52 patients hospitalized with PF over an approximately 4-year period ending in June 2022. The common diagnoses among the enrolled patients was interstitial pneumonia with autoimmune features (IPAF) (14/52), followed by Sjogren’s syndrome (pSS)-related ILD (6/52), IPF (5/52), and nonspecific interstitial pneumonia (NSIP) (4/52). Three patients each were diagnosed with systemic sclerosis (SSC)-related ILD, polymyositis-related ILD (PM-ILD), systemic lupus erythematosus (SLE-ILD), IgG4-related ILD (IgG4-RILD), rheumatoid arthritis (RA)-related ILD, and dust-related ILD (D-ILD). Two patients each were diagnosed with radiation pneumonitis (RP) and ANCA-associated vasculitis (AAV-ILD). One patient was diagnosed with hypersensitivity pneumonitis (HP)(Appendix Fig. 1A). Flow cytometry analysis of stored blood samples confirmed elevated levels of cytokines involved in the type 2 immune response as well as elevated IL-6, suggesting the activation of the type 2 immune response (Appendix Fig. 1B). Further analysis of additional lung tissue samples (from a total of 6 patients; Cases 1–6) showed that, compared with control samples from patients with bulla, patients with PF had higher expression of CD68 and βCGRP and lower expression of αCGRP (Appendix Fig. 2A). Further, immunofluorescence staining found dominant CD68^+^CD206^+^ M2-phenotype macrophage differentiation in the PF lung tissues (Fig. 1A, B).

Histopathological analysis of tissue available from a patient with PF showed proliferation of alveolar epithelial cells, presence of cellulose-like exudate in the alveolar cavity, a slightly widened alveolar septa with a large number of infiltrating lymphocytes and a small number of infiltrating neutrophils infiltrating, and no obvious atypical cells, consistent with alveolitis and PF (Fig. 1A, B, Appendix Fig. 1C). PET-CT of this patient revealed that the volume of the right lung was reduced. High density shadow with an uneven increase in metabolism was also noted in the right lung, in the posterior segment of the upper lobe tip of the left lung, and in the lower lobe of the left lung. A small amount of pleural effusion was visible on both sides (Appendix Fig. 1D–F). Lung high-resolution CT scan (HRCT) revealed an alveolar interstitial type with ground-glass opacity (GGO), patchy and strip-shaped density lesions with a fuzzy boundary and air bronchogram in the right lung (Appendix Fig. 1G–I). After 2 months of hormone therapy, reexamination of the lungs by HRCT showed a decrease in scattered patchy and strip lesions and only a few ground-glass shadows in the lower lobes of both lungs. In addition, the pleural effusion on both sides had been absorbed. These changes indicated that hormone therapy had improved the alveolitis and PF (Appendix Fig. 1J–L).

### Lungs of rats with αCGRP deficiency are similar to lungs in a BLM-induced model of PF

Based on our finding that αCGRP expression was decreased in PF lung tissue, we hypothesized that αCGRP deletion may induce PF. To study this, we evaluated lung tissue in a classic rat model of PF in which intratracheal administration of BLM is used to induce fibrosis [21], and we compared this with lung tissue from *Calca*^+/−^ rats. We confirmed that there was almost no αCGRP expression in *Calca*^+/−^ rats, whereas there was significantly higher expression of αCGRP in BLM and WT rats. Analysis of lung tissue from both models confirmed destruction of pulmonary tissue structure, fusion of pulmonary alveoli with inflammatory cells, and masses of broad-band or lamellar-shaped collagen fiber (Appendix Fig. 2B). In addition, infiltration of inflammatory cells in alveolar spaces and blood capillary of alveolar wall engorgement were also observed in the lung tissues of both BLM and *Calca*^+/−^ rats (Appendix Fig. 2B). Together, these data indicate that αCGRP can induce PF-like pathology in the rat lung.

### Abnormal immunocyte infiltration and apoptosis of alveolar epithelial cells in *Calca*-KO rats

**Fig. 2:**
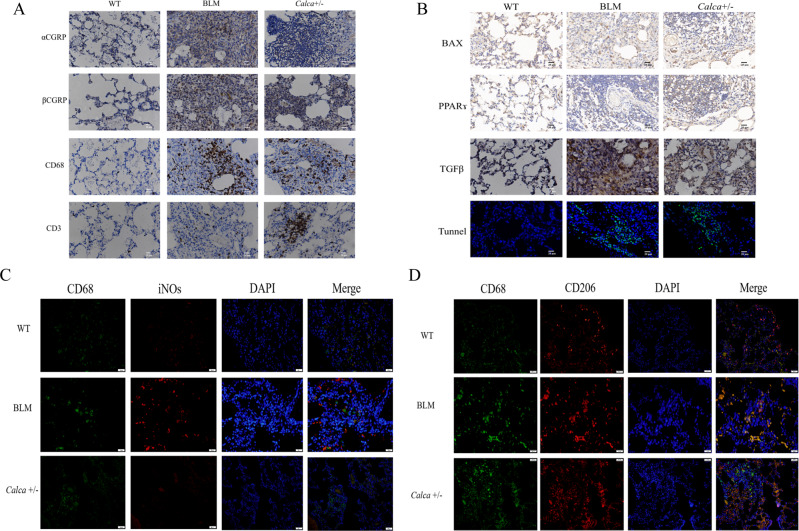
Immunohistochemistry results showed abnormal immunocytes aggregation with apoptosis of alveolar epithelial cells in *Calca*-KO rats. **A**, **B** The expression of βCGRP, BAX, PPARɤ, and TGFβ was higher in BLM and *Calca*^+/−^ rats compared to WT group (_400). There was abnormal expression of CD3, CD68, βCGRP, BAX, PPARɤ and TGFβ in the BLM group and *Calca*^+/−^ group. The TUNEL staining showed there were more positive alveolar epithelial cells in BLM group and *Calca*^+/−^ group than in WT control group (_200). Predominant CD68+CD206+M2 subtype differentiation was found in both *Calca*^+/−^ rats and BLM rats by immunofluorescence staining (In **C**, green represents CD68+ and red represents iNOS. In **D**, green represents CD68+ and red represents CD206+)(_200).

Next, we characterized the immune infiltrate and inflammatory markers in *Calca*^+/−^ rats to study whether αCGRP deficiency altered the immune microenvironment in ways that could induce PF development. The expression of βCGRP, BAX, PPARɤ, and TGFβ was higher in BLM and *Calca*^+/−^ rats compared to WT controls (Fig. 2A, B). In addition, CD3-positive and CD68-positive cells, indicating T lymphocytes and macrophages, respectively, were also increased in BLM and *Calca*^+/−^ rats compared to untreated WT rats. Further, immunofluorescence staining determined the infiltrating macrophages had differentiated to the M2 subtype in BLM-treated and *Calca*^+/−^ rats, as indicated by positive staining for CD68 and CD206 (Fig. 2C, D). In addition, we evaluated DNA damage using TUNEL staining, and we observed higher positive staining of alveolar epithelial cells in BLM -treated and *Calca*^+/−^ rats compared to untreated WT rats (Fig. 2A, B). Collectively, these experiments indicate that both inducing PF with BLM treatment as well as αCGRP deficiency result in high levels of apoptosis in the alveolar epithelial cells and an abnormal immune response consistent with PF characterized by infiltration of M2-differentiated macrophages and lymphocytes and by high inflammatory cytokine expression.

### αCGRP deficiency localizes PPARγto the nucleus and upregultes PPARγ signaling

**Fig. 3:**
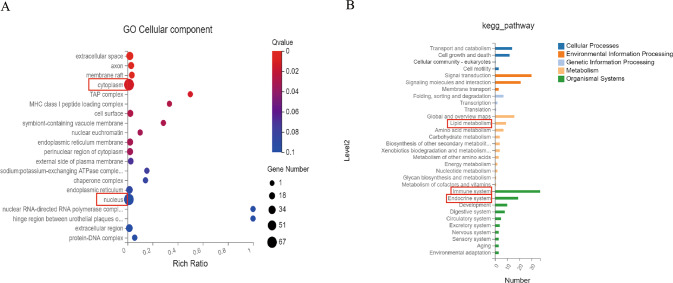
Pathway differences in lung tissue of *Calca*^+/−^ rats detected by RNA-seq. **A** The Gene Ontology (GO) analysis confirmed that the nucleus and cytoplasm expression of pathway proteins in *Calca*^+/−^ group was significantly different from that of WT group, which confirmed that *Calca*-KO affected nuclear translocation. **B** Kyoto Encyclopedia of Genes and Genomes (KEGG) pathways identified enrichment of pathways of the immune system, endocrine system and lipid metabolism in *Calca*^+/−^ compared to WT rats.

We analyzed gene expression in lung tissue from *Calca*^+/−^ and WT rats using RNA-seq, and subsequent Gene Ontology (GO) analysis showed that nuclear and cytoplasic expression pathways were significantly enriched in *Calca*^+/−^ rats compared to WT, indicating that αCGRP deficiency may induce specific proteins target intranuclear and cytoplasmic migration (Fig. 3A). Further, Kyoto Encyclopedia of Genes and Genomes (KEGG) analysis identified enrichment of pathways of the immune system, endocrine system and lipid metabolism in *Calca*^+/−^ compared to WT rats (Fig. 3B). These enriched pathways were further subdivided into the PPAR signaling pathway, TGFβ signaling pathway, Th1 and Th2 cell differentiation, apoptosis, fatty acid metabolism, fatty acid biosynthesis, fatty acid elongation, fatty acid degradation and cholesterol metabolism (Appendix Fig. 3). Based on the above results, we speculated that αCGRP deficiency may induce autoimmune disorders and lipid metabolic disorders, and that sustained activity of downstream effector molecules of PPAR signaling may induce PF. Thus, the following research focused on the relationship between the PPAR signaling pathway and lipid metabolism and its mechanism in PF.

**Fig. 4:**
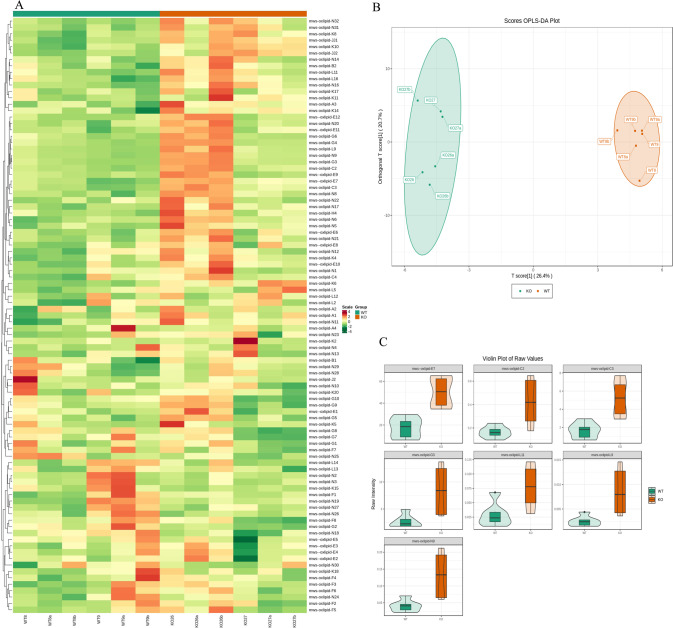
Analysis of metabolic differences. **A** There were differences in oxidized lipid group between the *Calca*^+/−^ group and the WT group. **B** The OPLS-DA model proved that the differences were obvious in these two groups. **C** After *Calca* knockout, the oxidized lipids were significantly upregulated. The enrichment analysis associated these dysregulated oxidized lipids with arachidonic acid metabolism, metabolic pathways, aldosterone synthesis and secretion, PPAR signaling, neuroactive ligand receptor interactions, serotonergic synapses and regulation of TRP channels by inflammatory mediators, bile secretion and eicosanic acid.

**Fig. 5:**
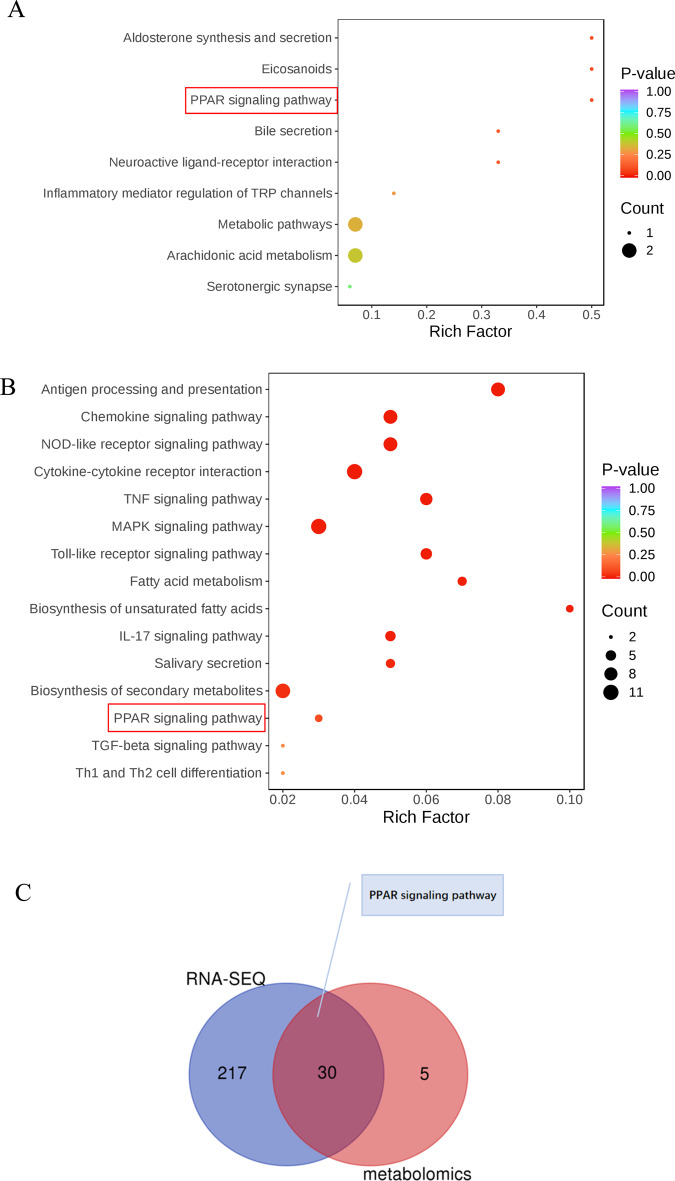
Combined analysis of transcriptome and oxidized lipid metabolomics in lung tissues confirmed the relationship between Calca knockout and PPAR pathway activation. KEGG pathways identified enrichment of PPAR signaling pathway in *Calca*^+/−^ compared to WT rats in both metabolomic datasets (**A**) and transcriptomic datasets (**B**). **C** Combined analysis of transcriptome and oxidized lipid metabolome showed that there were 30 common pathways between the two groups, including the PPAR pathway.

We next analyzed the lung tissues of *Calca*^+/−^ and WT rats using MS to determine whether αCGRP deficiency caused changes in metabolism (Fig. 4A, B). Indeed, the OPLS-DA model uncovered obvious differences between the two groups (Fig. 4B). Specifically, in *Calca*-KO rats, there was a significant increase in oxidized lipids, including (±)15-HEPE[(±)-15-hydroxy-5Z,8Z,11Z,13E,17Z-eicosapentaenoic acid], (±)12-HEPE[(±)-12-hydroxy-5Z,8Z,10E,14Z,17Z-eicosapentaenoic acid], (±)12-HETE[(±)12-hydroxy-5Z,8Z,10E,14Z-eicosatetraenoic acid], 14(S)-HDHA/HDoHE[14S-hydroxy-4Z,7Z,10Z,12E,16Z,19Z-docosahexaenoic acid], LTB4[5S,12R-dihydroxy-6Z,8E,10E,14Z-eicosatetraenoic acid], PDX[10(S),17(S)-dihydroxy-4Z,7Z,11E,13Z,15E,19Z-docosahexaenoic acid], and 13-HOTrE[13S-hydroxy-9Z,11E,15Z-octadecatrienoic acid]. The enriched oxidated lipid species were associated with arachidonic acid metabolism, aldosterone synthesis and secretion, PPAR signaling, neuroactive ligand receptor interactions, serotonergic synapses, regulation of TRP channels by inflammatory mediators, bile secretion, and eicosanic acid, suggesting that αCGRP deficiency results in an upregulation of these pathways in the lung (Figs. 4C and 5A).

We further combined KEGG pathway analysis of the transcriptome and oxidized lipid metabolome, and PPAR pathway signaling was significantly induced in both transcriptomic and metabolomic datasets in Calca-KO rats (Fig. 5A, B). Moreover, thirty common pathways, including the PPAR signaling pathway, were identified by combining analyses of the transcriptome and the oxidized lipid metabolome (Fig. 5C), strongly suggesting that αCGRP deficiency dysregulates PPAR pathway signaling in the lung.

**Fig. 6:**
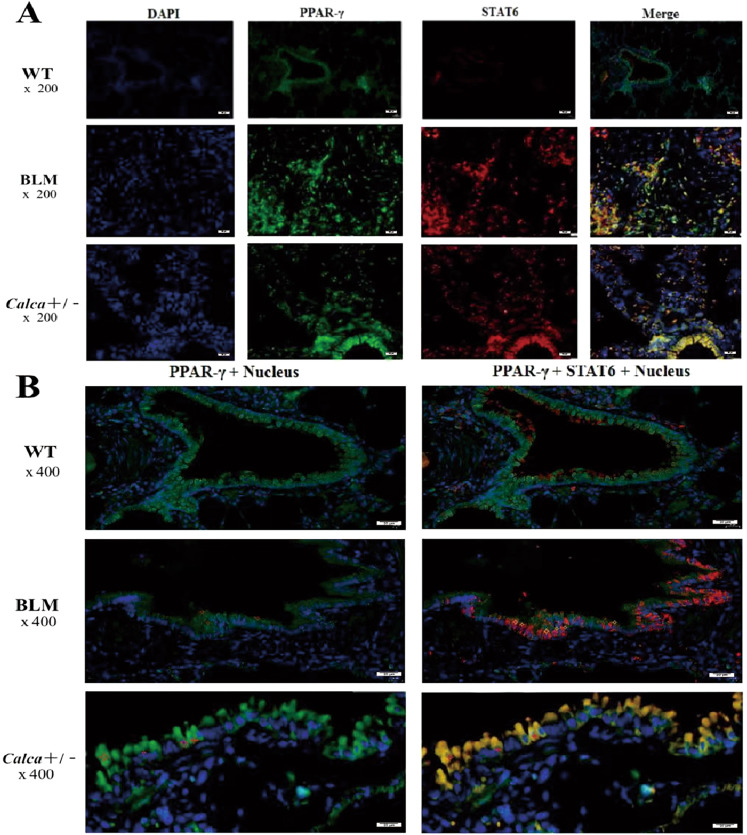
The co-expression of STAT6 and PPARγ in lung tissues of BLM induced pulmonary fibrosis mice and *Calca*^+/−^ mice. **A** Through nuclear staining and localization with DAPI, the co-expression of STAT6 and PPARγ has been found in the lung tissues of the BLM group and the *Calca*^+/−^ group. **B** The nuclear translocation of PPARγ in lung tissue of BLM group and *Calca*-KO rats which was synchronized with STAT6 in cytoplas and nuclear (marked with an asterisk in the picture).

Consistently, immunofluorescence analysis confirmed upregulated PPARγ nuclear translocation in the PF tissue of BLM-treated and *Calca*^+/−^ rats compared to untreated WT rats. Additionally, compared to untreated WT animals, the co-expression of STAT6 and PPARγ in the nucleus was increased in the lung tissues of the BLM-treated and *Calca*^+/−^ animals (Fig. 6A). Moreover, the nuclear translocation of PPARγ in lung tissue of BLM-treated and *Calca*^*+/−*^ rats was synchronized with STAT6 in the cytoplasmic and nuclear fractions (Fig. 6B).

## Discussion

PF is a chronic, pathological process and is the typical pathological manifestation of ILD. IPF is a chronic, progressive fibrosing interstitial pneumonia of unknown etiology that is associated with radiological and histologic features of usual interstitial pneumonia (UIP) and poor prognosis [4]. The official ATS/ERS/JRS/ALAT Clinical Practice Guideline published the physiological criteria for PPF in 2022, which differentiates PPF from IPF, prompting a paradigm shift toward an en bloc approach to treating PF with antifibrotic therapy [4]. However, a clinical trial reported a beneficial effect of antifibrotic medication in patients with PPF but not in patients with IPF [22, 23].

The plasticity, proximity, and ability to interact with the structural cells of the lung make macrophages a key cell type regulating PF. M1-like macrophages are pro-inflammatory, whereas M2-like macrophages are pro-fibrotic and associated with selective activation of wound healing [24]. Several recent studies have shown that M2 macrophage polarization plays a key role in the pathogenesis of tissue fibrosis [24–26]. Fibroblast foci consist of anti-apoptotic myofibroblasts and the extracellular matrix (ECM) they produce that lie beneath the alveolar epithelial cells (AECs). These foci persistently deposit collagen and represent active sites of fibrosis [27]. Thus, an intact superior alveolar cortex epithelium is protective against PF [27, 28]. Highly expressed pro-fibrotic factors secreted after M2 polarization during alveolitis can directly damage AECs and promote their apoptosis [29]. During M1-to-M2 polarization, activation of the STAT6/PPARγ and Th2 cytokines contribute to progression of PF [25]. However, there is a physiological negative feedback loop that inhibits STAT6/PPARγ signaling, and continuous pro-fibrotic signals during the early stages of lung injury are required for the initial injury to progress to PF [25].

We previously constructed a *Calca*^*+/−*^ rat model and demonstrated that the lung tissue of the *Calca*^*+/−*^ rats reproduced the pathological phenotype of fibrosis and M2 polarization, and we confirmed that abnormal αCGRP expression during PF development contributes to apoptosis of AECs [19]. However, how αCGRP interferes with M2 polarization and whether αCGRP deficiency further aggravates apoptosis of AECs and directly induces PF remains unclear. Here, we explored these questions in *Calca*^+/−^ rats and found that αCGRP deficiency reproduced the pathological phenotype of PF and resulted in M2 polarization of macrophages and apoptosis of AECs. Animal experiments also confirmed that the lungs of *Calca*^+/−^ rats exhibited the same PF-related changes and collagen deposition as the classical model of BLM-induced PF.

Analysis of human PF tissue found high expression of CD68^+^CD206^+^ M2 macrophages, low expression of CD68^+^ iNOs M1 macrophages, and high Th2 cytokine expression. The presence of CD3^+^ T lymphocytes, CD68^+^ macrophages, and CD68^+^CD206^+^ M2 macrophages as well as TGF-β expression were also higher in BLM-induced and *Calca*^+/−^ rats compared to untreated WT rats. We also found that the expression of αCGRP in the lung tissue of patients with PF was decreased. In our animal models, αCGRP expression in the lungs was higher in the WT group compared to the *Calca*^+/−^ group, where it was almost absent. M2-phenotype macrophages express high levels of various pro-fibrotic factors, such as TGFβ, EGF, Arg-1, IL-10, etc., which induce activation of Th2 cells [30]. The pro-fibrotic factors set up a positive feedback loop wherein M2-phenotype macrophages interact with Th2 cells, causing them to secrete cytokines such as IL-4 and IL-13 which can promote M2 polarization of macrophages [31]. Our results confirmed that M2-phenotype macrophages and Th2-dominated type 2 immune responses were activated in the lung tissue of PF patients and in BLM-induced PF in rats, as well as in *Calca*-KO rats, suggesting that αCGRP deficiency directly leads to M2 polarization of macrophages. Moreover, we confirmed apoptosis of AECs in BLM-treated and *Calca*^+/−^ rats, which was consistent with a trend of higher AEC apoptosis observed in patients with PF.

Compared with untreated WT rats, BLM-treated and *Calca*^+/−^ rats had higher expression of BAX and βCGRP in the lung tissue, suggesting that autoimmune dysregulation and apoptosis of epithelial cells contribute to the development of PF. Recently, some studies have shown that abnormal expression of CGRP promotes the development of PF, and that the down-regulation of CGRP during the development of PF can promote the release of TGFβ from M2 macrophages, resulting in a pathological transition from early alveolitis to PF [16]. Moreover, in vitro it has been demonstrated that exogenous CGRP can inhibit the proliferation and activation of TGFβ-induced lung fibroblasts as well as significantly inhibit the expression of TGFβ and collagen types I and III in a dose-dependent manner. These effects could be reversed using CGRP receptor blockers CGRP_8-37_ [32]. However, these studies did not investigate whether αCGRP or βCGRP played any role in the development of PF. In this study, we demonstrate that αCGRP protects AECs from apoptosis during the development of PF.

RNA-seq analyses showed that the lung tissue of *Calca*^+/−^ rats was characterized by immune dysfunction and increased nuclear localization of proteins, indicating αCGRP deficiency may induce specific proteins targeting intranuclear and cytoplasmic migration. KEGG analysis also identified enrichment of pathways related to the endocrine system and lipid metabolism in *Calca*^+/−^ compared to WT rats. We further identified these enriched pathways included the PPAR signaling pathway, TGFβ signaling pathway, Th1 and Th2 cell differentiation, apoptosis, fatty acid metabolism, fatty acid biosynthesis, fatty acid elongation, fatty acid degradation and cholesterol metabolism. PPARγ is a transcription factor and, in macrophages, PPARγ activates the type 2 immune response to promote the expression of IL-4 and IL-13. Early during the inflammatory response and alveolitis, PPARγ promotes TGFβ transcription, leading to massive apoptosis of AECs and development of PF; however, in advanced PF, PPARγ inhibits TGFβ transcription [33]. PPARγ also regulates lipid metabolism and the inflammatory responses in macrophages [34], and nuclear translocation of PPARγ can induce M2 macrophage polarization, regulate oxidative stress and fat metabolism, as well as control other facets of M2 macrophage biology [35, 36].

Metabolomic analysis identified 120 dysregulated oxidized lipids in the lung tissue of *Calca*^+/−^ rats. Compared with WT rats, LTB4, PDX, 12-HETE and 7 other species of oxidized lipid were enriched in *Calca*^+/−^ rats, and these correlated with dysregulated metabolic pathways, the PPAR signaling pathway neuroactive ligand receptor interactions, inflammatory mediators regulating TRP channels, and other pathways. Comparison of dysregulated gene expression and metabolic pathways identified 30 pathways that were altered in *Calca*^+/−^ rats, including the PPAR pathway. Studies have shown that 12-HETE can be used as a biomarker of liver fibrosis and elevated 12-HETE is associated with good prognosis in patients with liver fibrosis [37]. Metabolomic analysis of regulatory lipid mediators in sputum from patients with cystic fibrosis revealed that abnormal lipid mediators, including 12-HETE, were associated with lung function [38]. Leukotrienes (LTs) belong to a large family of lipid mediators and are related to various inflammatory states such as asthma and rheumatoid arthritis. The G protein-coupled receptor CysLT2, a LT receptor, is involved in inflammatory pain, and CysLT2 mRNA is positive for transient receptor potential vanilloid 1 (TRPV1), which releases CGRP and is highly colocalized with neurons [39]. Recent studies have also found that high levels of LTB4, a key endogenous molecule that induces the neutrophil inflammatory response, are associated with PF [40], and LTB4 biosynthesis blockade is now a potential strategy for the treatment of IPF and cystic PF [41, 42]. Therefore, we speculate that upregulated lipid oxidation in *Calca*-KO animals might be an important contributor to the development of PF in these models.

Finally, we confirmed the co-expression of STAT6 and PPARγ in the pulmonary fibrosis tissues of BLM-treated and *Calca*^+/−^ rats, as well as synchronization of the cytoplasmic and nuclear entry of PPARγ and STAT6, thus confirming that αCGRP deficiency promotes PPARγ nuclear translocation. Studies have shown that inhibition of the STAT6/PPARγ pathway in liver fibrosis can reduce M2 macrophage polarization and delay fibrosis progression [43]. Combined with our data, this suggests that loss of αCGRP can induce autoimmune dysfunction and PF through the PPAR signaling pathway as well as maintain the activity of downstream effector molecules of the PPAR signaling, leading to PF progression.

Notably, the mechanism by which αCGRP deficiency specifically promotes nuclear translocation of PPARγ to promote apoptosis of AECs remains unknown. It may be that the metabolites of αCGRP react with pro-fibrotic metabolites in the body, thereby reducing the damage of pro-fibrotic products to AECs. One study showed that PDX could reverse the phenotypic transformation of epithelial-to-mesenchymal transition (EMT) in lung tissue of mice with PF and also had a protective effect on renal fibrosis [44, 45], but our metabolomics study found that αCGRP deficiency caused the upregulation of PDX expression. We speculate this may be a compensatory increase in the body, but the specific mechanism is unknown. The mechanism by which αCGRP exerts an antifibrotic effect needs to be further clarified, but our metabolomic data suggest that its regulation of LTB4, 12-HETE and other oxidized lipids may be biologically relevant in this regard.

In summary, αCGRP may inhibit the nuclear translocation of PPARγ during the development of pulmonary fibrosis and reduce the activation of downstream pro-fibrotic type 2 immune responses, so as to protect the integrity of the alveolar epithelial layer and inhibit the differentiation of fibroblasts and collagen deposition.

## Conclusion

*Calca* deficiency induced severe apoptosis in AECs of fibrotic lung tissues. αCGRP may inhibit the nuclear translocation of PPARγ, thereby inhibiting M2 macrophage polarization and the release Th2-dominated type 2 immune response cytokines. Our data also show that αCGRP affects the survival and functional status of AECs by regulating the PPARγ pathway and the expression of downstream proteins that protect from PF by promoting proliferation, inhibiting apoptosis, and preventing continuous, ineffective tissue repair.

## Supplementary information

supplementary legends

Appendix1

Appendix2

Appendix3
