# Supplementary material for: αCGRP deficiency aggravates pulmonary fibrosis by activating the PPARγ signaling pathway
Source: Genes Immun. 2023 May 25;24(3):139–48. doi: 10.1038/s41435-023-00206-x (PMC10266974; doi:10.1038/s41435-023-00206-x)
Supplement: Supplementary file 1 — supplementary legends [file 41435_2023_206_MOESM1_ESM.docx]

**Appendix 1 Clinical records of 53 patients with PF patients**

A: The commonest disease type in PF was interstitial pneumonia with autoimmune features (IPAF) (14/52)，followed by Sjo¨gren’s syndrome (pSS)-related ILD (6/52), IPF (5/52), nonspecific interstitial pneumonia (NSIP) (4/52), systemic sclerosis (SSC)-related ILD(3/52), polymyositis related ILD (PM-ILD) (3/52), systemic lupus erythematosus (SLE-ILD) (3/52), IgG4-related ILD (IgG4-RILD) (3/52), rheumatoid arthritis (RA)-related ILD (3/52), dust-related ILD (D-ILD)(3/52), ANCA-associated vasculitis (AAV-ILD) (2/52), radiation pneumonitis (RP)(2/52) and hypersensitivity pneumonitis (HP)(1/52)；B：Abnormal activation of type 2 immune response was founded in the serum of PF patients by flow cytometry , and IL-6 was a dominant factor in Th2 cytokines；C：The pathology of percutaneous lung biopsy of case3 showed that there were proliferation of alveolar epithelial cells, cellulose like exudate in the alveolar cavity, slightly widened alveolar septa with a large number of lymphocytes and a small number of neutrophils infiltrating; D-F: PET-CT revealed that the volume of the right lung was reduced. High density shadow with uneven increase of metabolism could be seen in the right lung, the posterior segment of the upper lobe tip of the left lung, and the lower lobe of the left lung. At the same time, a small amount of pleural effusion can be seen on both sides (April 10, 2019) (case3); G-I: Lung HRCT revealed an alveolar interstitial type that was evaluated for the presence and distribution of ground-glass opacity(GGO), patchy and strip shaped density lesions with a fuzzy boundary and the air filled bronchial sign in the right lung. A small amount of pleural effusion also could be found on both sides, mainly in the right lung (April 23, 2019)(case3); J-L: After 2 months of hormone therapy, reexamination of lung CT showed that scattered patchy and strip lesions were less than before, and pleural effusion on both sides had been absorbed. Only a few ground glass shadows could be seen in the lower lobes of both lungs, indicating that changes in alveolitis and pulmonary fibrosis were significantly better than before (July 1, 2019) (case3).

**Appendix 2: Establishment and verification of lung fibrosis in patients and rats**

A: Compared with bulla group, the expression of CD68 and βCGRP was increased and the expression of αCGRP was decreased in patients of PF; B:There were fewer inflammatory cells, less collagen deposition and fibrosis in WT group. Compared with the WT group, H&E and Masson's staining in the BLM group showed typical changes of PF, such as destroyed lung tissue structure and masses of lamellar shaped collagen fiber. *Calca-*KO could reproduce the typical pathological changes of PF, such as a part of pulmonary alveoli fused with inflammatory cells and masses of collagen fiber.

**Appendix 3: The enriched pathways identified by KEGG analysis**

The PPAR signaling pathway, TGFβ signaling pathway, Th1 and Th2 cell differentiation, apoptosis, fatty acid metabolism, fatty acid biosynthesis, fatty acid elongation, fatty acid degradation and cholesterol metabolism were identified by KEGG analysis.
