# Supplementary figures and images for: αCGRP deficiency aggravates pulmonary fibrosis by activating the PPARγ signaling pathway
Source: Genes Immun. 2023 May 25;24(3):139–48. doi: 10.1038/s41435-023-00206-x (PMC10266974; doi:10.1038/s41435-023-00206-x)

## Slide 1
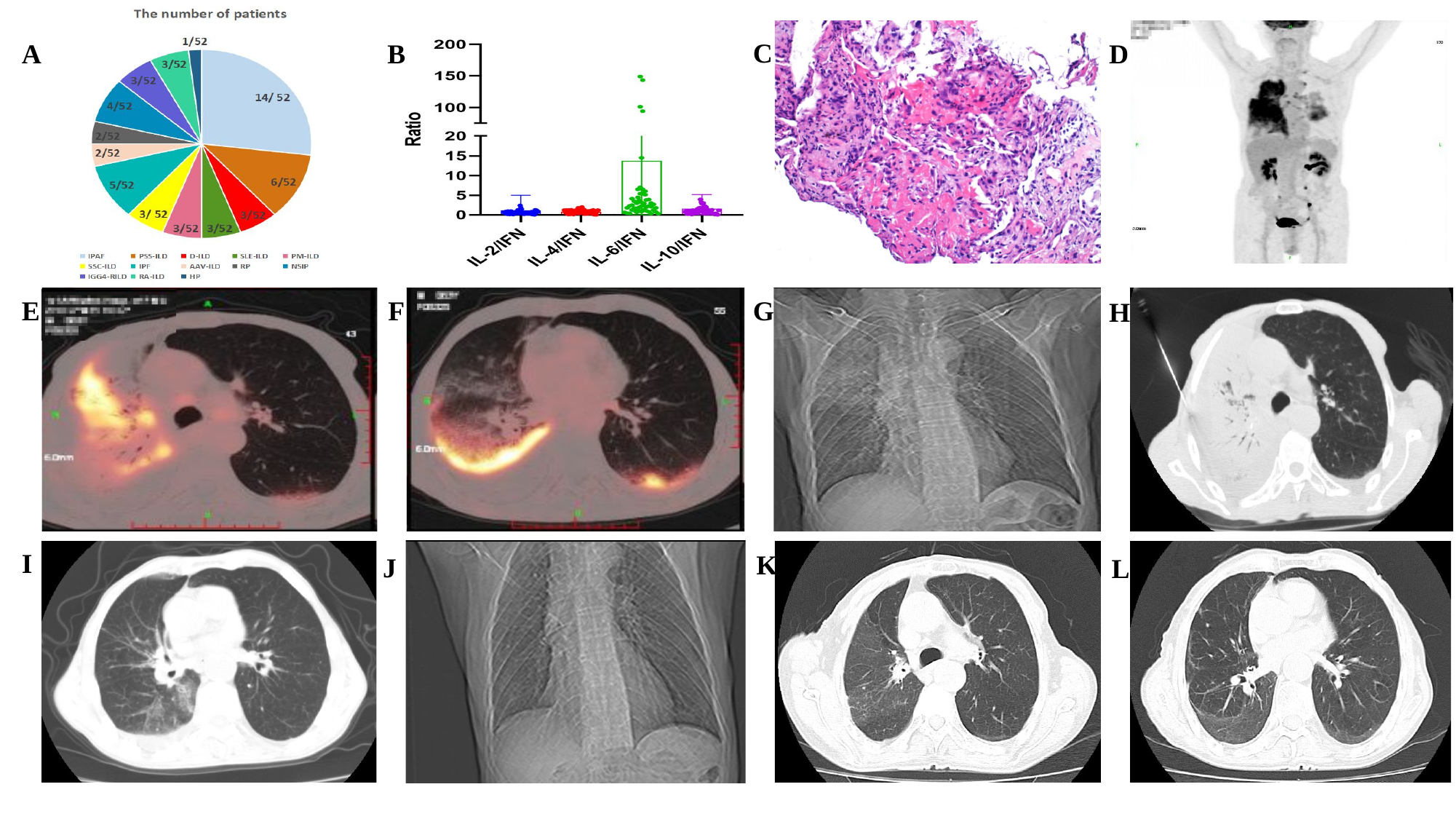

C
A
B
D
E
F
G
H
I
K
J
L

Supplement: Supplementary file 2 — Appendix1 [file 41435_2023_206_MOESM2_ESM.pptx]

## Slide 1
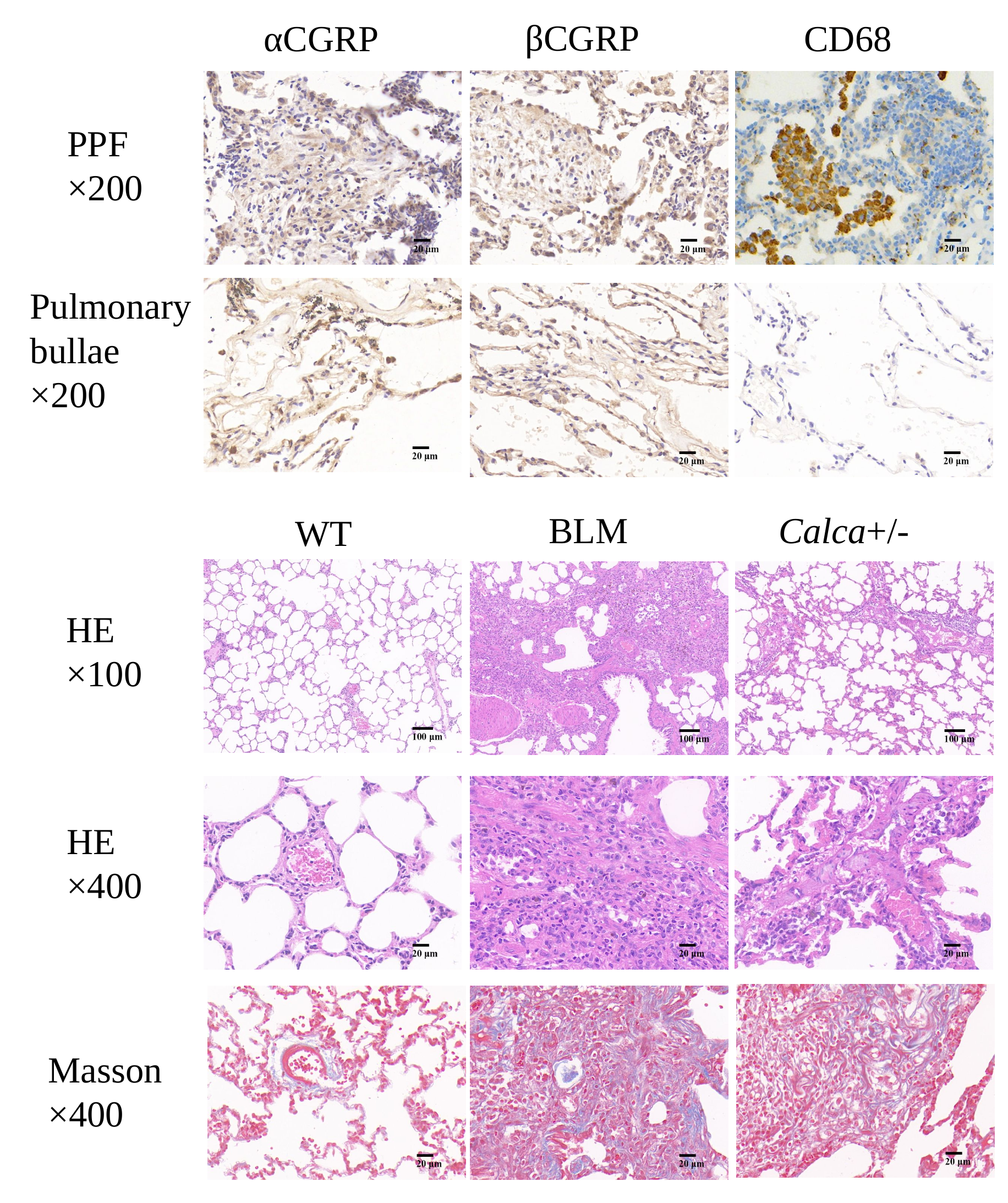

βCGRP
αCGRP
 CD68
PPF
×200
Pulmonary bullae
×200
BLM
Calca+/-
WT
HE
×100
HE
×400
Masson
×400

Supplement: Supplementary file 3 — Appendix2 [file 41435_2023_206_MOESM3_ESM.pptx]

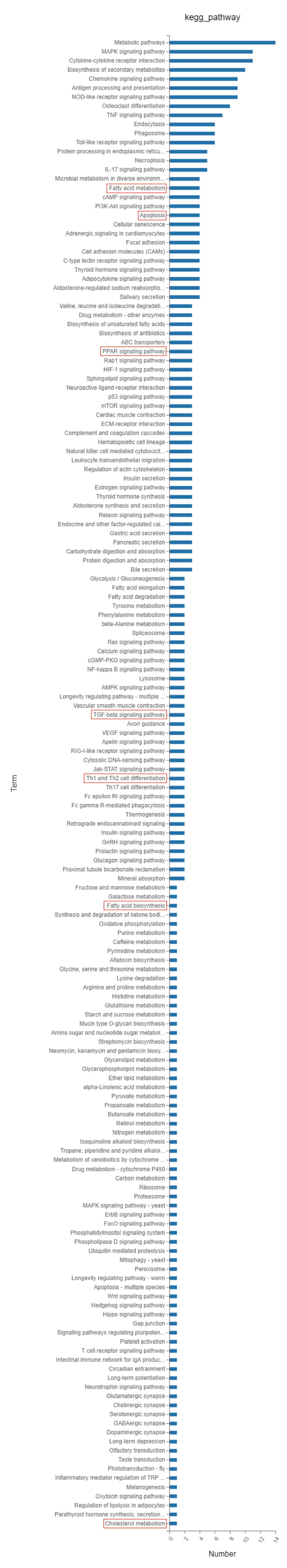

Supplement: Supplementary file 4 — Appendix3 [file 41435_2023_206_MOESM4_ESM.pdf]
